# Nonhuman Primate Welfare: From History, Science, and Ethics to Practice

**DOI:** 10.1017/awf.2023.42

**Published:** 2023-06-19

**Authors:** Giulia Ciminelli, David Massey, Kate Stupples, Claire Witham

**Affiliations:** 1 Institute of Biosciences, Newcastle University; 2 Centre for Macaques, MRC Harwell, Medical Research Council

This is a very comprehensive book about the welfare of non-human primates covering a broad range of topics and edited by Lauren M Robinson and Alexander Weiss.

The book is broken down into five sections:

- History of non-human primates in captivity and primate welfare in different settings;
- Assessing non-human primate welfare;
- Non-human primate housing and husbandry;
- Individual differences, application, and improvement of non-human primate welfare; and
- Biomedical research, ethics and legislations surrounding non-human primate welfare.

Within each section the editors have invited contributions from a range of backgrounds and views with the majority focused on laboratory or zoo primates. There are a few chapters on primates kept as pets and entertainers and primates housed in sanctuaries. The majority of the chapters are well-written and engaging with just the occasional chapter that seemed out of place in its section or the book.

As this is a comprehensive book, we each reviewed a separate section (as reviewers we have experience in laboratory primates, zoo primates, colony management and primate welfare). Within the first four sections we found chapters that stood out to us as reviewers as being particularly useful for primate welfare in a range of settings.

Part 1 covers the history of primates in captivity and an overview of primate welfare of primates in laboratory, zoo and pet/entertainment settings as well as an update on primate welfare in South America. The chapter on ‘Using Primates in Captivity: Research, Conservation, and Education’ (Mark J Prescott) stands out as being particularly useful. This covers a range of research settings including the use of primates in biomedical research, studies at zoos and fieldwork with wild primates and provides a good set of guidelines for the use of primates in research that will be useful to anyone planning studies involving primates.

Part 2 provides a range of tools for assessing primate welfare whilst making it clear there is no one tool that covers all aspects of welfare. These tools include assessing behaviour, cognitive bias tests, physiological measures and questionnaires. Of these, the chapter on assessing behaviour (Corrine K Lutz and Kate C Baker) provides an overview of the different types of behaviour to assess and suggests some potential interventions for abnormal behaviours. This chapter will be relevant for a wide range of settings and gives a good introduction for students of primate welfare.

Part 3 gives a comprehensive overview of the challenges of housing primates in captivity. It is good to see a chapter on breeding colony management (James C Ha and Adrienne F Sussman) since there are unique challenges associated with breeding primates in groups within biomedical research compared with pair housing/small groups in laboratories. The chapters covering housing and husbandry in biomedical research (Kristine Coleman *et al*.) and zoos (HL Farmer *et al*.) give a useful overview of the challenges of meeting the needs of primates in captivity.

Part 4 has chapters on personality, social relationships, enrichment and training and their application to primate welfare. The enrichment chapters will be useful for animal care staff. The chapter on sociality (Beisner *et al*.) reviews the importance of social relationships and gives an important overview of the costs and benefits of sociality which is important for anyone who is housing primates in social groups. There is also a useful chapter on the research benefits of improving welfare in captive primates (Steven J Schapiro and Jann Hau) which will help laboratory primate care staff argue for improvements to primate welfare in their institutions.

Part 5 feels like a miscellaneous collection compared to the other sections and includes the arguments for and against using primates in research, an example of a specific primate model, ethical frameworks and an overview of different regulations around the world.

Overall, we think this book will appeal to a wide range of people who work with and study primates although the cost of the book may be a barrier to some.

